# Meiotic Studies in Some Species of Tribe Cichorieae (Asteraceae) from Western Himalayas

**DOI:** 10.1155/2014/673456

**Published:** 2014-11-13

**Authors:** Raghbir Chand Gupta, Henna Goyal, Vijay Singh, Rajesh Kumar Goel

**Affiliations:** ^1^Department of Botany, Punjabi University, Patiala 147002, India; ^2^Department of Pharmaceutical Sciences and Drug Research, Punjabi University, Patiala 147002, India

## Abstract

The present paper deals with meiotic studies in 15 species belonging to 6 genera of the tribe Cichorieae from various localities of Western Himalayas. The chromosome number has been reported for the first time in *Hieracium crocatum* (2*n* = 10) and *Lactuca lessertiana* (2*n* = 2x = 16). Further, intraspecific variability has been reported for the first time in *H. umbellatum* (2*n* = 2x = 10 and 2*n* = 6*x* = 54), *Tragopogon dubius* (2*n* = 2*x* = 14 and 2*n* = 4*x* = 28), and *T. gracilis* (2*n* = 2*x* = 14). The chromosome report of 2*n* = 2*x* = 10 in *Youngia tenuifolia* is made for the first time in India. Maximum numbers of the populations show laggards, chromosome stickiness, and cytomixis from early prophase to telophase-II, leading to the formation of aneuploid cells or meiocytes with double chromosome number. Such meiotic abnormalities produce unreduced pollen grains and the reduced pollen viability.

## 1. Introduction

The tribe Cichorieae (also known as Lactuceae) encompasses 95 genera and ca. 2500 species, primarily in temperate to subtropical zones of the Northern Hemisphere [1]. Members of the tribe are characterized by very important uniform characteristics, such as homogamous ligulate capitula and the presence of milky latex.

The presently investigated species are also known to have medicinal uses, such as* Hieracium crocatum* to cure gastric troubles,* Lactuca dolichophylla* to cure constipation,* L. macrorhiza* used as an ingredient to cure stomach ache,* L. serriola* to treat ailments of the urinary tract, and* Taraxacum officinale* used as diuretic and laxative [2–4].

Chromosome studies are valuable determinants in studying evolution. Many workers have studied the cytology of Indian Asteraceae, including members of tribe Cichorieae. Cytological studies on the family from Lahaul-Spiti area, an ecologically very fragile cold desert area of Western Himalayas, are almost lacking. As an attempt to fill this lacuna, the present investigations have been undertaken.

## 2. Materials and Methods

### 2.1. Plant Material

Exploratory surveys were made during the years from 2009 to 2013 in selected localities (Table 1) of Himachal Pradesh (Kullu and Lahaul-Spiti Districts). The cytologically worked-out plants were identified using regional floras and compared with the specimens deposited at the Herbarium of Botanical Survey of India, Northern Circle, Dehra Dun. The voucher specimens (Table 1) were deposited in the Herbarium, Department of Botany, Punjabi University, Patiala (PUN). 

### 2.2. Meiotic Studies

For meiotic chromosome counts, unopened floral buds of suitable sizes were fixed in a freshly prepared Carnoy's fixative (mixture of alcohol, chloroform, and glacial acetic acid in a volume ratio 6 : 3 : 1) for 24 h. These were subsequently transferred to 70% alcohol and stored in refrigerator at 4°C until used for meiotic analysis. Meiocytes were prepared by squashing the developing anthers and stained with acetocarmine (1%). Chromosome number was determined at diakinesis/M-I/II/A-I/II from freshly prepared slides with light microscope Olympus. 500−600 pollen mother cells were analyzed for meiotic behaviour at different stages, metaphase-I/II (M-I/II), anaphase-I/II (A-I/II), and telophase-I/II (T-I/II). 

### 2.3. Pollen Grain Analysis

Pollen fertility was estimated through stainability tests using glycerol-acetocarmine (1 : 1) mixture and aniline blue (1%). Up to 450−800 pollen grains were examined for pollen fertility and size frequencies. Well-filled pollen grains with stained nuclei were taken as apparently fertile while shriveled and unstained pollens were counted as sterile. 

### 2.4. Photomicrographs

Photomicrographs from the freshly prepared desirable slides having clear chromosome counts, dyads, triads, tetrads, and pollen grains were taken with a digital imaging system of Leica QWin.

## 3. Results

### 3.1. Chromosome Number

**Figure 1:**
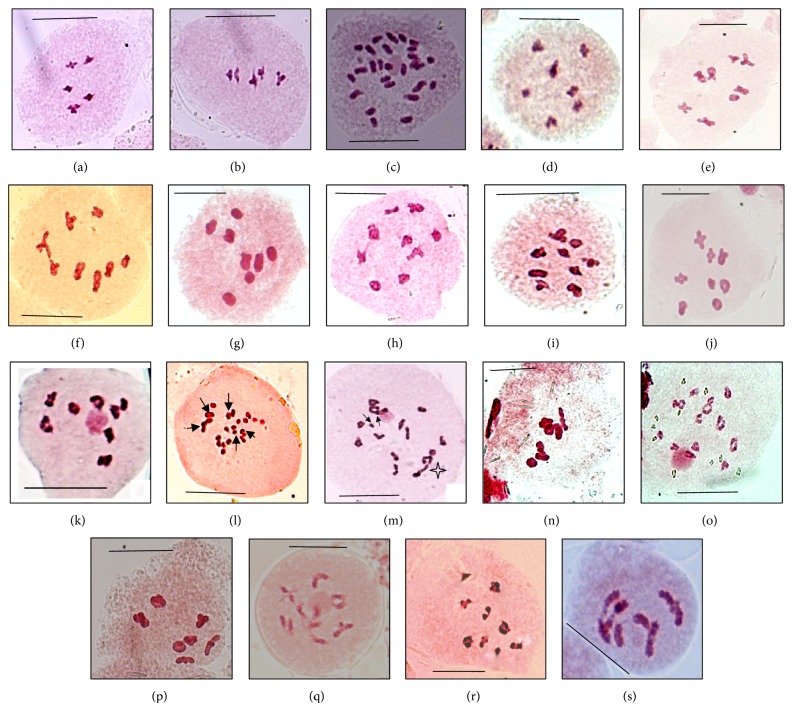
(a)* Hieracium crocatum*, PMC showing 5II at M-I. (b-c)* H. umbellatum*: (b) diploid cytotype, PMC showing 5II at M-I, (c) hexaploid cytotype, PMC showing 27II at diakinesis. (d)* Lactuca dissecta*, A PMC showing 8II at M-I. (e)* L. dolichophylla*, PMC showing 8II at M-I. (f)* L. lessertiana*, PMC showing 8II at M-I. (g)* L. macrorhiza*, PMC showing 8II at M-I. (h)* L. sativa*, PMC showing 9II at M-I. (i)* L. serriola*, PMC showing 9II at M-I. (j)* Prenanthes brunoniana*, PMC showing 8 bivalents at M-I. (k–m)* Taraxacum officinale*: (k) diploid cytotype, PMC of diploid cytotype showing 8II at diakinesis, (l) triploid cytotype, PMC showing 5II + 14I at M-I (bivalents with arrow), and (m) tetraploid cytotype, PMC showing 1IV + 13II + 2I (univalents with arrow and quadrivalent with star). (n-o)* Tragopogon dubius*: (n) diploid, PMC showing 7II at M-I, (o) tetraploid cytotype, PMC showing 14II at diakinesis. (p)* T. gracilis*, PMC showing 7II at M-I. (q)* Youngia glauca*, PMC showing 7II at diakinesis, (r)* Y. japonica*, PMC showing 8II at M-I. (s)* Y. tenuifolia*, A PMC showing 5II at diakinesis. (M-I: metaphase-I, scale: 10 *μ*m, IV: quadrivalent, II: bivalent, I: univalent.)

*Hieracium crocatum Bunge ex. Ledeb.* The present species revealed the diploid cytotype (2*n* = 10, Figure 1(a)), which is a first ever chromosome report for the species.

**Figure 2:**
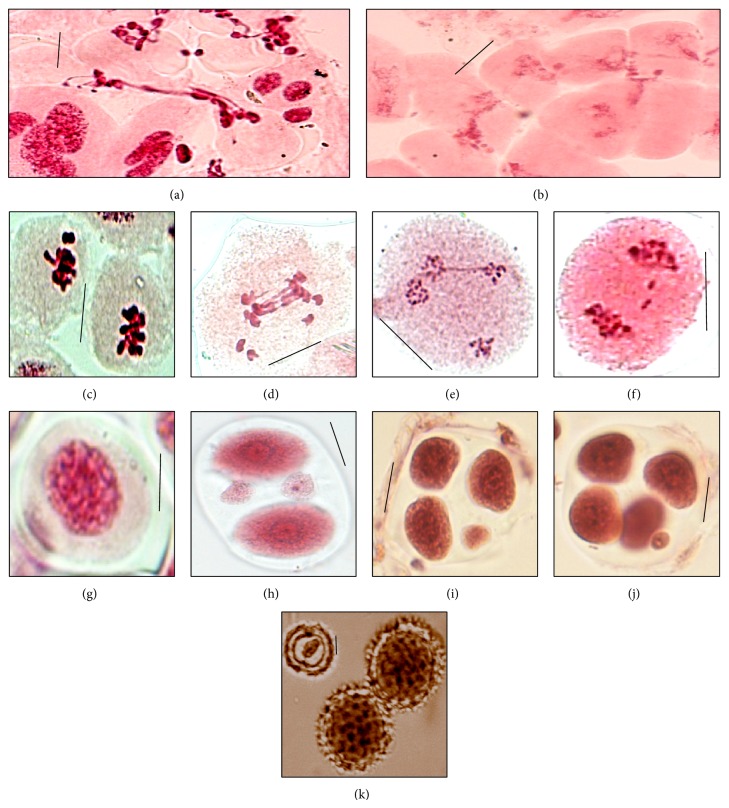
(a-b) PMC showing transfer of chromatin material in* Taraxacum officinale* and* Hieracium umbellatum*, respectively. (c) PMC at metaphase-I showing chromatin stickiness in tetraploid cytotype of* T. officinale*. (d) PMC showing late disjunction at anaphase-1 (*arrowed*). (e) PMC at telophase-II showing chromatin bridges (*arrowed*). (f) PMC at anaphase-I showing chromatin laggards (*arrowed*) in triploid cytotype of* T. officinale*. (g) Monad. (h) Diad with micronuclei. (i) Triad with micronucleus. (j) Tetrad with micronucleus. (k) Heterogeneous sized fertile and sterile (*arrowed*) pollen grains. (Scale: 10 *μ*m, IV: quadrivalent, II: bivalent, I: univalent.)

*H. umbellatum L.* Both the cytotypes, 2*n* = 10 (Figure 1(b)) and 2*n* = 54 (Figure 1(c)), are the new records from the world, although the species is already known to have 2*n* = 18 [5] and 2*n* = 27 [6] from outside India. Polyploid cytotypes show some enlargement in vegetative and floral characters (Table 2).

*Lactuca dissecta D. Don.* The present report (2*n* = 16, Figure 1(d)) is in line with many previous reports from India [7–10] and abroad [11].

*L. dolichophylla Kitam.* The present chromosome report of 2*n* = 16 (Figure 1(e)) is in line with many previous reports from India [7, 8, 10, 12]. 

*L. lessertiana (Wall. ex DC) C. B. Clarke.* The present meiotic studies reveal a diploid cytotype (2*n* = 16, Figure 1(f)) which is a first ever chromosome report for the species.

*L. macrorhiza (Royle) Hook. f.* The present chromosome report of 2*n* = 16 (Figure 1(g)) is already confirmed by many workers [8, 12, 13] from India. There is no chromosome record from abroad for the species.

*L. sativa L.* Meiotic analysis of the species reveals the diploid cytotype (2*n* = 18, Figure 1(h)), which is in conformity with the previous works of Chatterjee and Sharma [14] and Gupta and Gill [15].

*L. serriola L.* The present chromosome report (2*n* = 18, Figure 1(i)) is confirmed by many workers from India [7, 12, 15, 16].

*Prenanthes brunoniana C. B. Clarke.* The present chromosome report of 2*n* = 16 (Figure 1(j)) is in conformity to only previous report from Garhwal, Uttarakhand, by Shetty [8].

*Taraxacum officinale L.* The present meiotic investigation revealed three cytotypes, 2*n* = 2*x* = 16 (Figure 1(k)), 2*n* = 3*x* = 24 (Figure 1(l)), and 2*n* = 4*x* = 32 (Figure 1(m)). Both diploid and tetraploid cytotypes are common and are reported by many workers [17]. Gupta et al. [13], besides triploid cytotype, also reported some other cytotypes, that is, 2*n* = 26, 27, 32, 38, and 40. Morphologically, the tetraploids do not show any robust and gigas effect due to polyploidy (Table 2), but they certainly show a lot of variation in shape of leaves.

*Tragopogon dubius Scop.* Both the cytotypes, 2*n* = 14 (Figure 1(n)) and 2*n* = 28 (Figure 1(o)), are varied cytotypes at world level. The species is reported earlier with 2*n* = 24 by Koul and Gohil [18] and Mehra and Remanandan [12] from Kashmir Himalayas. From outside India, the species is known to have 2*n* = 12, 24, and 36 [5]. Morphologically, tetraploid cytotype does not show any gigas effect as compared to diploid (Table 2).

*T. gracilis D. Don.* The present chromosome report (2*n* = 14, Figure 1(p)) is a varied chromosome count for the species at world level. Earlier, Mehra and Remanandan [12] reported diploid cytotype with 2*n* = 12 from the Western Himalayas.

*Youngia glauca Edgew.* The present cytological investigation reveals the diploid cytotype (2*n* = 14, Figure 1(q)), which is a varied chromosome report. Earlier, there is a maiden cytological report of 2*n* = 16 [19] from Kinnaur valley.

*Y. japonica (L.) DC.* The present chromosome report (2*n* = 16, Figure 1(r)) confirms the earlier reports from different localities of India and abroad [20].

*Y. tenuifolia (Wild.) Babcock and Stebbins.* This chromosome report of 2*n* = 10 (Figure 1(s)) is for the first time reported from India. The same number is frequently reported from abroad [21, 22].

### 3.2. Meiotic Abnormalities

Meiotic abnormalities have been recorded in almost all the studied populations of different species in the form of cytomixis, chromatin stickiness, unoriented bivalent, bridges, laggards, or multipolarity at different stages of meiosis (Tables 3 and 4, Figures 2(a)–2(k)). From the data, triploid cytotype of* Taraxacum officinale* shows the highest percentage of chromatin transfer from prophase-I to telophase-II (Table 2, Figure 2(a)). Cytomixis usually led to the formation of pollen mother cells (PMCs) with different chromosome numbers and even empty PMCs in some cases (Figure 2(b)), as is evident in hexaploid cytotype of* Hieracium umbellatum*. Chromatin stickiness (partial or often complete clumping of bivalents) is found in 16.28 per cent of PMCs and unoriented bivalents were seen in 13.79 per cent of PMCs of tetraploid cytotype of* T. officinale* (Table 3, Figure 2(c)). The present investigation also reveals abnormal meiosis in the form of chromosomal laggards (maximum in triploid cytotype of* T. officinale*) and bridges (maximum percentage in tetraploid cytotype of* H. umbellatum*) at anaphase-I/II and telophase-I/II (Figures 2(e)-2(f)). These meiotic abnormalities led to the abnormal microsporogenesis and the formation of heterogeneous sized pollen grains (Table 4, Figures 2(g)–2(k)) and also affect pollen viability (Table 1).

## 4. Discussion

### 4.1. Chromosome Number

#### 4.1.1. Tribe Cichorieae

The ancestral basic numbers in the tribe are *x* = 4, 5, and 9, as suggested by Turner et al. [23]. But Stebbins et al. [24] proposed *x* = 9 as the base number for Cichorieae (Asteraceae in general), which is supported by Tomb et al. [25], with the other numbers (*x* = 3–9) derived through phylogenetic reduction through chromosomal aberrations, particularly translocation. 

*Hieracium L.* About 360 species are cytologically known with 94 species being diploid and 152 triploid and 149 species are tetraploid and rarely possess aneuploidy (7 spp.). The genus forms agamic complex and is considered monobasic on *x* = 9. But, the present study reveals another cytotype (2*n* = 10), suggesting a new base number (*x* = 5) in the genus. 

*Lactuca L.* A total of 150 species are known taxonomically, of which chromosome numbers for 87 species overall and 14 species from India are known. The chromosome number in the genus varies in the range of 2*n* = 10–48 and is polybasic on *x* = 5, 8, 9, and 17, of which *x* = 9 is the most dominant number. 

*Prenanthes L.* Twenty-two species in the genus are known cytotaxonomically, including 1 from India. The most common base number is *x* = 8 represented with 19 species, including diploids (16 species) and tetraploids (3 species). However, the intraspecific polyploids are not available in the genus. Besides, *x* = 9 is also present in 3 species that are diploid. Hence, the genus is proposed to be dibasic on *x* = 8 and 9. 

*Taraxacum L.* The genus is very complex, reinforcing the reason of having 347 cytologically (including 10 species from India) worked-out species. The chromosome numbers vary in the range of 2*n* = 8–64, the most common of which is 2*n* = 3*x* = 24 (230 spp.) on *x* = 8, followed by diploid (47 spp.) on the same base number. Genus is reported to have a series of base numbers on *x* = 4, 6, 8, 9, and 11, but only *x* = 8 is known to have well-developed polyploid races (2*x*–6*x*). Intraspecific polyploidy is also known to occur in *x* = 9 and 11 (1 species each). 

*Tragopogon L.* 75 species in the genus are cytologically known, with chromosome number in the range of 2*n* = 12–36, almost all based on *x* = 6. The overall polyploidy in the genus is 26.6% (20 spp.), out of which 14 species show intraspecific polyploidy. The variable chromosome number of 2*n* = 14 is found in 4 species (including the present data) and 2*n* = 28 in only one species (from the present data). 

*Youngia L.* A total of 35 species are taxonomically known, cytology is reported for only 14 species (including 5 from India), with 9 species showing polyploid cytotypes (3*x*, 4*x*, and 6*x*). The chromosome numbers reported so far are 2*n* = 10, 15, 16, 20, 24, 32, and 42, out of which 2*n* = 10 (43.7%) is the most common followed by 2*n* = 16 (31.2%). The genus is polybasic (*x* = 5, 7, and 8), of which *x* = 5 is most common.

### 4.2. Meiotic Abnormality

The phenomenon of inter-PMC migration of chromatin/chromosome between/among the contiguous meiocytes through cytomictic channels is termed as cytomixis (coined by Gates [26]). However, the phenomenon has been reported for the first time in gymnosperms by Arnoldy [27] and subsequently in angiosperms by Koernicke [28]. Since that time, cytomixis has been reported in a large number of plants [29]. Transfer of chromatin or chromosomes may take place through such inter-PMC cytomictic channels [30–32]. Some workers reported cytomixis to be more prevalent in polyploids than their diploid counterparts [33, 34]. Occasionally, either hypoploid meiocytes [35–37] or enucleated meiocytes or meiocytes with a hyperploid number of chromosomes have been reported [30, 37–39]. It is very much clear that the enucleated meiocytes die, but hypo- and hyperploid meiocytes could lead to the formation of gametes with variable chromosome number and size. Cytomixis is considered as a process of evolutionary significance because it results in change in gametic chromosome numbers [30, 40]. Chromosome stickiness also results in the formation of fragmented chromatin. This chromatin stickiness, late or nondisjuncting bivalents, and chromosomal laggards seem to be responsible for chromosomal bridges [41]. All these meiotic abnormalities consequently assert an effect on microsporogenesis, leading to the formation of monads, dyads, triads, or polyads with or without micronuclei, which ultimately produce heterogeneous sized (large and small) fertile pollen grains and reduced pollen fertility. The size difference may be due to the formation of unreduced gametes (2*n*), which may produce plants with higher ploidal level through polyploidization (for review, see [42–45]).

As observed in the presently investigated data, the chromatin rearrangement due to meiotic abnormalities is considered the base of inter- or intraspecific diversity. Further, it provides a catalogue for studying different evolutionary trends such as breeding system or polyploidy and hybridization.

## Figures and Tables

**Table 1:** The table showing details on taxon, geographical coordination, accession number, average plant height, flowering-fruiting period, chromosome number (2*n*), ploidal level (*x*), and pollen fertility of tribe Cichorieae (Asteraceae) from Western Himalayas.

Taxon	Locality with altitude (m) and geographical coordinates	Accession number (PUP^*^)	Average plant height (cm)	Flowering-fruiting period	Chromosome number (2*n*)	Ploidal level (*x*)	Pollen fertility (%)
*Hieracium crocatum* Bunge ex Ledeb.	Trilokinath, Lahaul (H.P.), 2760 32°42′0′′ N, 76°41′0′′ E	52760	42 ± 6.4	June–September	10^‡^	2*x*	88.4
*H. umbellatum* L.							
(P1)	Jispa, Spiti (H.P.), 3142 32°38′0′′ N, 77°10′0′′ E	52763	42 ± 2.3	June–September	10^‡^	2*x*	91.0
(P2)	Keylong, Lahaul (H.P.), 3080 32°34′48′′ N, 77°1′48′′ E	52774	46 ± 7.2	June–September	54^‡^	6*x*	82.1
*L. dissecta* D. Don.	Hadimba, Temple, Manali, 2438 32°14′32′′ N, 77°11′14′′ E	58562	16 ± 4.3	June–September	16	2*x*	88.1
*L. dolichophylla* Kitamura	Sissu, Lahaul (H.P.), 3130 32°29′0′′ N, 77°7′0′′ E	57504	20 ± 5.2	June–September	16	2*x*	79.2
*L. lessertiana* C. B. Clarke	Batal, Spiti (H.P.), 3890 32°21′28′′ N 77°37′10′′ E	52776	32 ± 7.2	June–September	16^‡^	2*x*	70.2
*L. macrorhiza* (Royle) Hook. f.	Chotadhara, Spiti (H.P.) 3800	57510	25 ± 4.6	June–September	16	2*x*	71.3
*L. sativa* L.	Hadimba, Temple, Manali, 2438 32°14′32′′ N, 77°11′14′′ E	58534	55 ± 2.4	March–November	18	2*x*	87.0
*L. serriola* L.	Jispa, Spiti (H.P.), 3200 32°38′0′′ N, 77°10′0′′ E	58535	55 ± 2.5	April–October	18	2*x*	76.0
*Prenanthes brunoniana* C. B. Clarke	Tandi, Keylong (H.P.), 2573 32°34′40′′ N, 77°1′36′′ E	52764	80 ± 13.1	June–August	16	2*x*	90.1
*Taraxacum officinale* L.							
(P1)	Marhi, Manali (H.P.), 3320 32°20′56′′ N, 77°13′4′′ E	52687	27.1 ± 3.2	March–November	16	2*x*	86.2
(P2)	Keylong, Lahaul (H.P), 3080 32°34′48′′ N, 77°1′48′′ E	52693	8.3 ± 7.9		32	4*x*	73.6
(P3)	Kibber, Spiti (H.P.), 4205 32°19′54′′ N, 78°0′32′′ E	57491	17.2 ± 1.6		24	3*x*	56.3
*Tragopogon dubius* Scop.							
(P1)	Keylong, Lahaul (H.P), 3080 32°34′48′′ N, 77°1′48′′ E	57503	50 ± 2.5	June–September	14^‡^	2*x*	83.0
(P2)	Lossar, Spiti (H.P.), 4079 32°24′49′′ N, 77°49′11′′ E	57505	35 ± 7.3	June–September	28^‡^	4*x*	90.3
*T. gracilis* D. Don	Koksar, Lahaul (H.P.), 3160 32°41′37′′ N, 77°23′54′′ E	52768	25.2 ± 4.9	June–September	14^‡^	2*x*	79.2
*Youngia glauca* Edgew.	Zingzingbar, Lahaul (H.P.), 4270 32°47′30′′ N, 77°19′28′′ E	52773	73 ± 5.3	July-August	14	2*x*	83.0
*Y. japonica* (L.) DC.	Hadimba, Temple, Manali, 2438 32°14′32′′ N, 77°11′14′′ E	57500	42 ± 4.7	June–September	16	2*x*	78.2
*Y. tenuifolia* (Wild.) Babc.	Jispa, Spiti (H.P.), 3200 32°38′0′′ N, 77°10′0′′ E	52771	15 ± 4.2	June–September	10^†^	2*x*	87.2

^*^Herbarium, Punjabi University, Patiala. ^‡^First ever chromosomal report. ^†^First cytotype report from India.

**Table 2:** Morphological comparison of different cytotypes of tribe Cichorieae from Western Himalayas.

Taxon/voucher data	Chromosome number (2*n*)	Leaf size (mean ± SD)	Shape of leaf	Flower colour	Average number of capitula/plant	Stomatal size (*µ*m) (mean ± SD)	Pollen size (*µ*m) (mean ± SD)	Stomatal index (%)
*Hieracium umbellatum *								
P1—52763	10	4.0 ± 0.2 × 1.0 ± 0.3	Entire	Yellow	17	23.21 ± 0.4 × 31.54 ± 2.4	16.75 ± 0.7 × 20.1 ± 1.2	33.33
P2—52774	54	7.0 ± 1.2 × 1.5 ± 0.5	Serrate-toothed	Yellow	22	29.80 ± 2.1 × 34.26 ± 1.7	15.0 ± 0.56 × 20.0 ± 0.9	23.07
*Taraxacum officinale *								
P1—52687	16	20.0 ± 0.6 × 1.9 ± 0.9		Yellow	3	17.6 ± 0.4 × 14.5 ± 0.9	18.75 ± 0.4 × 20.62 ± 0.8	28.57
P2—52693	32	5.9 ± 1.2 × 2.6 ± 0.6		Yellow	4	18.75 ± 0.3 × 20.1 ± 0.4	18.98 ± 1.4 × 19.75 ± 1.6	23.07
P3—57491	24	6.9 ± 0.6 × 1.9 ± 0.7		Purple	6	18.30 ± 1.3 × 18.98 ± 2.8	18.75 ± 2.4 × 23.0 ± 2.5	30.0
*Tragopogon dubius *								
P1—57503	14	15 ± 0.5 × 0.6 ± 0.3	Linear	Yellow	23	22.19 ± 0.5 × 27.54 ± 0.7	23.0 ± 1.3 × 12.0 ± 0.8	28.5
P2—57505	28	15 ± 0.5 × 0.9 ± 0.3	Linear lanceolate	Yellow	17	18.36 ± 1.2 × 15.25 ± 0.8	22.75 ± 0.7 × 20.11 ± 1.4	16.66

^*^SD: standard deviation.

**Table 3:** Data on cytomixis and meiotic course in the studied populations of tribe Cichorieae from Western Himalayas.

Accession number	Cytomixis		Meiotic course			
	PMCs involved (% age)	Number of PMCs involved (%)	PMCs with chromosomal stickiness at M-I (%)	PMCs with unoriented bivalents at M-I (%)	PMC with bridges (at A-I, II/T-I, II) (%)	PMCs with laggards (at A-I, II/T-I, II) (%)
52760	0.8 (10/125)	1-2	3.10 (4/129)	7.20 (9/125)	10.08 (12/114)	10.56 (13/123)
52763	3.96 (8/126)	1-2	5.50 (6/109)	2.30 (3/130)	4.31 (6/139)	1.55 (2/129)
52774	11.71 (13/111)	2–4	11.57 (14/121)	13.79 (16/118)	26.95 (38/141)	10.44 (14/134)
52764	2.4 (3/125)	1-2	4.0 (5/125)	—/—	4.0 (5/125)	—/—
58562	5.4 (6/111)	1-2	3.17 (4/126)	2.70 (3/111)	—/—	—/—
57504	—/—	0	0.82 (1/121)	1.80 (2/111)	4.50 (5/111)	0.82 (1/121)
52776	—/—	0	1.66 (2/120)	—/—	—/—	—/—
57510	2.77 (4/144)	1-2	3.47 (5/144)	2.0 (3/144)	—/—	—/—
58534	—/—	0	—/—	—/—	—/—	—/—
58535	1.0 (1/97)	1-2	11.34 (11/97)	6.1 (6/97)	7.21 (7/97)	2.06 (2/97)
52687	4.35 (5/144)	2-3	5.50 (6/109)	8.39 (11/131)	3.84 (8/130)	10.08 (12/114)
52693	1.78 (2/112)	1-2	7.20 (9/125)	—/—	3.10 (4/129)	3.84 (8/130)
57491	29.91 (35/117)	2–6	16.28 (21/129)	13.79 (16/116)	12.5 (17/136)	10.44 (14/134)
57503	2.43 (3/123)	1-2	2.30 (3/130)	—/—	—/—	—/—
57505	1.72 (2/160)	1-2	1.37 (2/145)	2.38 (3/126)	1.16 (2/125)	3.25 (4/123)
52768	—/—	0	—/—	—/—	—/—	—/—
52773	0.88 (1/113)	1-2	3.53 (4/113)	1.76 (2/113)	5.30 (6/113)	1.76 (2/113)
57500	—/—	0	—/—	—/—	—/—	—/—
57771	1.61 (2/124)	1-2	1.62 (2/123)	—/—	—/—	—/—

Figures in parenthesis denote observed number of abnormal PMCs in the numerator and total PMCs observed in the denominator.

**Table 4:** Data on abnormal microsporogenesis on different accession of tribe Cichorieae from Western Himalayas.

Taxon/accession numbers	Monads	Dyads		Triads		Tetrads	
	WM (%)	WM (%)	WMN (%)	WM (%)	WMN (%)	WM (%)	WMN (%)
58533	—/—	—/—	0.97 (1/103)	—/—	2.91 (3/103)	—/—	96.11 (99/103)
52760	2.75 (3/109)	0.91 (1/109)	2.75 (3/109)	—/—	2.75 (3/103)	—/—	90.82 (99/109)
52763	—/—	—/—	1.80 (2/111)	2.70 (3/111)	1.80 (2/111)	3.60 (4/111)	90.09 (100/111)
52774	1.5 (2/130)	0.76 (1/130)	2.30 (3/130)	3.07 (4/130)	2.3 (3/130)	3.8 (5/130)	86.15 (112/130)
52764	—/—	—/—	1.6 (2/120)	—/—	—/—	—/—	98.3 (118/120)
58562	—/—	—/—	—/—	—/—	—/—	—/—	100 (123/123)
57504	—/—	0.95 (1/105)	—/—	1.90 (2/105)	2.8 (3/105)	1.90 (2/105)	92.38 (97/105)
52776	—/—	—/—	1.66 (2/121)	—/—	4.13 (5/121)	—/—	95.04 (115/121)
57510	—/—	0.80 (1/124)	1.61 (2/124)	—/—	4.03 (5/124)	1.61 (2/124)	91.93 (114/124)
58534	—/—	1.05 (1/95)	—/—	—/—	—/—	—/—	99.2 (139/140)
58535	—/—	1.72 (2/116)	4.31 (5/116)	2.58 (3/116)	4.31 (5/116)	5.17 (6/116)	81.89 (95/116)
52687	—/—	1.5 (2/130)	0.76 (1/130)	0.76 (1/130)	2.30 (3/130)	1.5 (2/130)	93.07 (121/130)
52593	—/—	—/—	0.8 (1/125)	2.4 (3/125)	0.8 (1/125)	1.6 (2/125)	94.4 (118/125)
57491	2.29 (2/87)	4.59 (4/87)	1.14 (1/87)	2.29 (2/87)	3.44 (3/87)	11.49 (10/87)	72.41 (63/87)
57503	—/—	—/—	—/—	—/—	—/—	—/—	100 (121/121)
57505	—/—	—/—	0.8 (1/125)	—/—	1.6 (2/125)	0.8 (125)	96.8 (121/125)
52768	1.66 (2/121)	4.13 (5/121)	0.82 (1/121)	4.13 (5/121)	0.82 (1/121)	0.82 (1/121)	87.60 (106/121)
52773	—/—	0.86 (1/115)	2.60 (3/115)	—/—	2.60 (3/115)	1.73 (2/115)	92.17 (106/115)
57500	1.66 (2/121)	0.82 (1/121)	0	0.82 (1/121)	0.82 (1/121)	13.22 (16/121)	82.64 (100/121)
52771	—/—	—/—	3.10 (4/129)	0.75 (1/129)	6.97 (9/129)	8.52 (11/129)	80.60 (104/117)

Figures in parenthesis denote observed number of abnormal PMCs in the numerator and total number of PMCs observed in denominator; WMN: without micronuclei, WM: with micronuclei.
